# An Interactive Website to Reduce Sexual Risk Behavior: Process Evaluation of TeensTalkHealth

**DOI:** 10.2196/resprot.3440

**Published:** 2015-09-02

**Authors:** Sonya S Brady, Renee E Sieving, Loren G Terveen, BR Simon Rosser, Amy J Kodet, Vienna D Rothberg

**Affiliations:** ^1^ Division of Epidemiology & Community Health University of Minnesota School of Public Health Minneapolis, MN United States; ^2^ University of Minnesota School of Nursing Minneapolis, MN United States; ^3^ Division of General Pediatrics and Adolescent Health Department of Pediatrics University of Minnesota Medical School Minneapolis, MN United States; ^4^ Department of Computer Science and Engineering University of Minnesota Institute of Technology Minneapolis, MN United States

**Keywords:** adolescent, sexual health, technology, intervention studies, evaluation

## Abstract

**Background:**

Different theoretical frameworks support the use of interactive websites to promote sexual health. Although several Web-based interventions have been developed to address sexual risk taking among young people, no evaluated interventions have attempted to foster behavior change through moderated interaction among a virtual network of adolescents (who remain anonymous to one another) and health professionals.

**Objective:**

The objective was to conduct a summative process evaluation of TeensTalkHealth, an interactive sexual health website designed to promote condom use and other healthy decision making in the context of romantic and sexual relationships.

**Methods:**

Evaluation data were obtained from 147 adolescents who participated in a feasibility and acceptability study. Video vignettes, teen-friendly articles, and other content served as conversation catalysts between adolescents and health educators on message boards.

**Results:**

Adolescents’ perceptions that the website encouraged condom use across a variety of relationship situations were very high. Almost 60% (54/92, 59%) of intervention participants completed two-thirds or more of requested tasks across the 4-month intervention. Adolescents reported high levels of comfort, perceived privacy, ease of website access and use, and perceived credibility of health educators. Potential strategies to enhance engagement and completion of intervention tasks during future implementations of TeensTalkHealth are discussed, including tailoring of content, periodic website chats with health educators and anonymous peers, and greater incorporation of features from popular social networking websites.

**Conclusions:**

TeensTalkHealth is a feasible, acceptable, and promising approach to complement and enhance existing services for youth.

## Introduction

Young people account for approximately half of unintended pregnancies [1] and contracted sexually transmitted infections (STIs) [2] in the United States each year. Interventions that provide complete and accurate information to adolescents, as well as motivation and behavioral skills to negotiate condom use with partners, have demonstrated success with respect to increasing consistency of condom use [3-11]. Such interventions explicitly or implicitly target constructs from the Information-Motivation-Behavioral skills (IMB) model of human immunodeficiency virus (HIV) risk reduction, which posits that risk-reduction information, motivation, and behavioral skills are fundamental determinants of risk behavior change [12]. Information relevant to STI prevention and motivation to reduce risk are posited to exert direct effects on condom use and to exert indirect effects through activation of risk-reduction behavioral skills. Motivation to engage in condom use is thought to be a function of several cognitions, including attitudes toward condoms, perceived social norms, and perceived personal vulnerability to STIs. Behavioral skills include sexual communication and negotiation skills. Although IMB-based sexual health interventions have succeeded in promoting condom use, sizable percentages of youth who receive interventions subsequently engage in inconsistent condom use and contract STIs [5-8]. This highlights the need for novel interventions that build on the IMB model.

Interventions that aim to increase and sustain consistency of condom use among youth appear to be fighting an uphill battle. It is normative for condom use to decline within and across successive relationships [13-15]. Motivation to use condoms may be undermined by a variety of factors, including equation of condom use with lack of intimacy and trust in one’s partner [16-20]; perceptions that it is only the man’s responsibility to obtain and carry condoms [21] and that possession of condoms is evidence of promiscuity [18,19,22]; greater concern for prevention of pregnancy than STIs [13,20,21,23-25]; reliance on hormonal contraceptives versus dual forms of contraceptives that include condom use [24,26]; involvement in a physically or emotionally abusive relationship, which may lead to inequities in power with respect to sexual decision making [27]; and sexual behavior in the context of substance use, which may lead to other forms of risk taking, including inconsistent condom use [28,29]. Regardless of the pathway by which motivation to use condoms wanes, inconsistent condom use can become habitual. It is thus important for adolescents to establish consistent patterns of condom use and other healthy behaviors early in their relationships and to challenge thoughts that may lead to normative declines in condom use.

Highly interactive, moderated websites are an ideal setting to foster health protective thoughts and behaviors. Adolescents feel comfortable using the Internet to obtain health information [30,31] and to express concerns to peers [32] and health professionals [33]. Across a variety of age groups, peer-based interventions to promote health commonly use websites as a forum to interact [34,35]. In the United States, an estimated 84% of youth aged 8 to 18 years have Internet access in their homes (78% among African American youth; 75% among Hispanic youth) [36]. Approximately 70% of youth go online daily and nearly 75% have created a social networking site profile [36]. Although it is difficult to predict changes in technology and youth culture [37], it is likely that websites will remain highly accessible to youth (eg, websites can be made mobile-compatible using a microbrowser). Private interactive websites can be designed to mimic the most appealing aspects of public social media websites, while also maximizing privacy and confidentiality for users. Intervening with youth on a public social media website may preclude discussion about sensitive personal information because privacy and security settings are owned and controlled by someone other than the health provider’s or researcher’s institution [38].

Different theoretical frameworks support the use of interactive websites to promote sexual health. Although behavior change theories—such as the IMB model of HIV risk reduction [12]—are useful in targeting areas for intervention, an individual’s adoption of recommended behavior change may depend on the successful application of communication theory [39]. An intervention must not only be instructive, but persuasive [39-41]. Health communications must promote attention by being engaging and interesting, while also balancing arousal with comfort, promote understanding by being clear, and promote acceptance by appearing relevant and credible and by appealing to cognitions that influence motivation [41,42]. The Internet allows users to provide immediate feedback on whether different types of health communications are attended to, understood, and accepted. To be effective, health communications must also lead to little counterarguing (thoughts that inhibit agreement with an advocated position) [39]. Some adolescents are skeptical of information provided by authority figures [43]. In addition, adults are unlikely to guess the factors that will increase adolescents’ attention to, understanding of, and acceptance of messages promoting health protective behavior [44]. Thus, it is critical to adopt methodology in which adolescents can provide guidance on the content and delivery of health communications [44]. Design-based research—a systematic, but flexible, methodology aimed to improve educational practices and outcomes—is particularly well suited to the development of technology-enhanced learning environments [45]. It involves iterative analysis, design, development, and implementation of an educational product. A fundamental feature is ongoing collaboration between researchers and recipients of the intervention to ensure that inquiry and practice are responsive to a group’s needs [45]. The Internet can facilitate ongoing interaction between adolescents and health educators and allow for interventions that are responsive to the potentially changing needs of individual adolescents over time.

**Figure 1:**
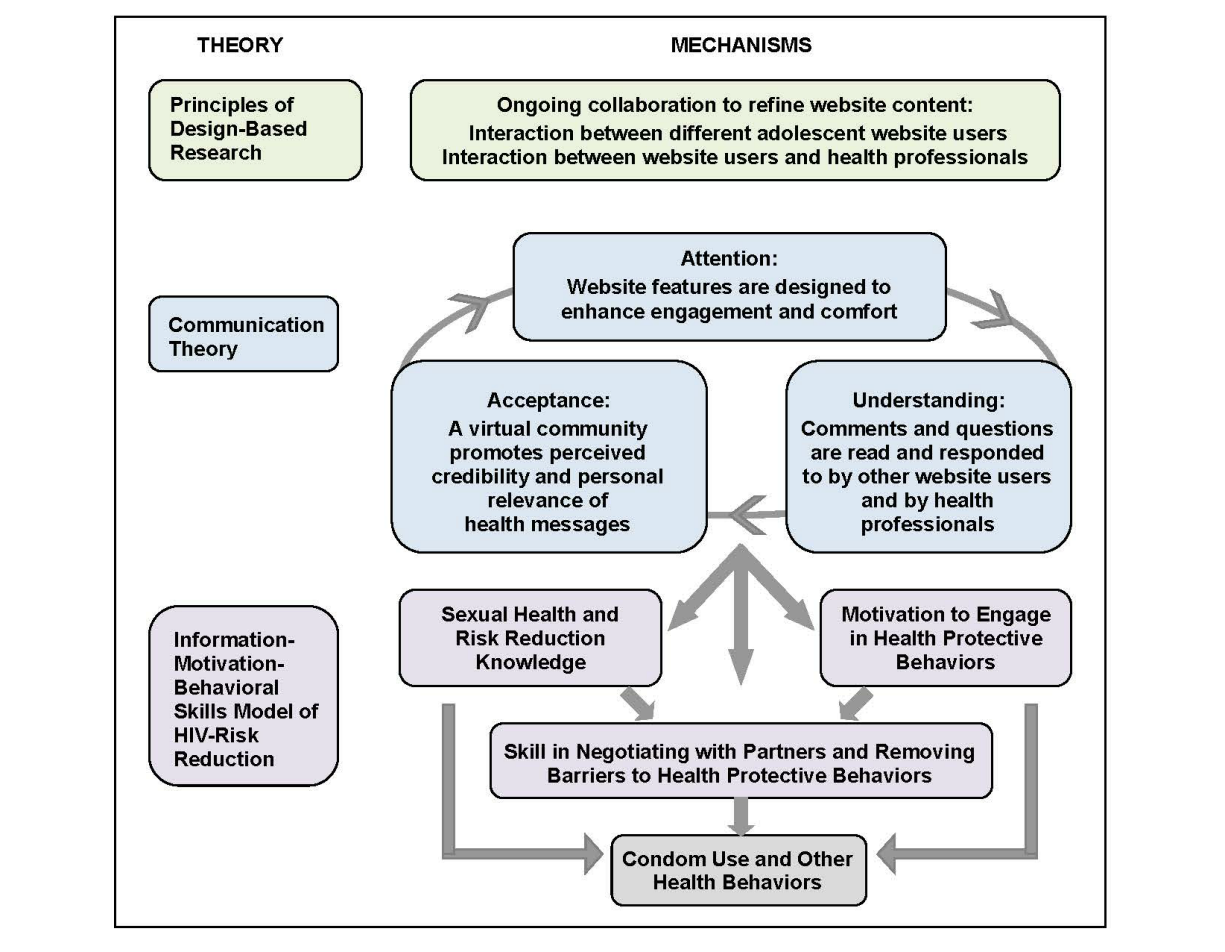
Mechanisms of behavior change with respect to condom use and other health behaviors.

Although several Web-based interventions have been developed to address sexual risk taking among young people [46-52], no evaluated interventions have attempted to foster behavior change through moderated interaction between a virtual network of adolescents, who remain anonymous to one another, and health professionals. TeensTalkHealth is an interactive Web-based intervention designed to promote condom use and other healthy decision making in the context of romantic and sexual relationships. As depicted in Figure 1, the TeensTalkHealth intervention aims to increase condom use and other health behaviors through targeting constructs of the IMB model of HIV risk reduction [12]. TeensTalkHealth provides information, motivation, and behavioral skills to decline risk behaviors, negotiate health protective behaviors, and build healthy relationships. Constructs of communication theory and principles of design-based research guide the TeensTalkHealth approach with respect to behavior change. Video vignettes, teen-friendly articles, and other content are designed to promote attention to, understanding of, and acceptance of health-promoting messages. These standardized components of the TeensTalkHealth intervention serve as conversation catalysts between adolescent website users and health educators, who have the opportunity to interact with one another via message board discussions, a key feature of the website intervention. Health educators and adolescent peers can read and respond to comments and questions posted by individual adolescents, which may serve to enhance the perceived credibility and personal relevance of health-promoting messages. Consistent with principles of design-based research, adolescents are consulted in the initial design of the website and planning of intervention content. Importantly, adolescent participants are able to shape website content as the intervention unfolds. Adolescent website users can interact with one another and with health professionals to shape the content of message board discussions and, potentially, the order in which health professionals decide to feature predeveloped content on the website. This may enhance attention to, understanding of, and acceptance of health-promoting messages. The TeensTalkHealth intervention approach has 3 primary advantages: (1) support—interaction with peers and health educators as part of a virtual community can provide opportunities for learning and support; (2) convenience—access to content and interaction with others can occur on an ongoing basis, including times of greatest convenience and/or need; and (3) anonymity—individuals who are anonymous to one another may be more comfortable, which may increase candor, relevance of website content, and participant engagement.

This research examines the feasibility and acceptability of delivering a confidential, peer-based sexual health intervention through the Internet, which may lead to the expansion of treatments and services for youth. This paper describes a summative process evaluation [53] of the TeensTalkHealth website intervention. Evaluation data were obtained from 147 adolescents who participated in a study to determine the feasibility and acceptability of the website intervention and assessment methods. Data were collected across a 4-month intervention period and 2-month follow-up period. Findings can be used to guide further development of the TeensTalkHealth intervention and other interactive websites that aim to promote healthy decision making among adolescents.

## Methods

### Study Procedure and Participants

The University of Minnesota Institutional Review Board approved this research. A federal certificate of confidentiality was obtained to protect sensitive data obtained from adolescents.

The principal investigator (first author) faxed letters and/or sent emails of introduction to executive directors of community-based teen clinics and principals of public and charter schools, followed initial modes of contact by one voicemail message if necessary, and met with interested staff to explain the purpose of the study and answer questions. Three of 5 approached community clinics became recruitment partners; 1 of the remaining 2 clinics planned to become a partner, but closed before recruitment began. Three of 17 approached schools became recruitment partners.

Recruitment took place between January and October 2011. Clinic staff were asked to distribute and collect recruitment flyers from all adolescents aged 14 to 18 years seeking services. At 2 school sites, research staff gave presentations about healthy relationships or sexual health during class, briefly described the study, and distributed and collected flyers immediately afterwards. The third school site distributed flyers to age-eligible students through email. Flyers contained a brief description of the study, including the potential to earn up to US $140 across a 6-month period. Adolescents were asked to fill out nonidentifying demographic information on flyers (age, sex, race/ethnicity). Those who were interested in the study were asked to add contact information.

**Figure 2:**
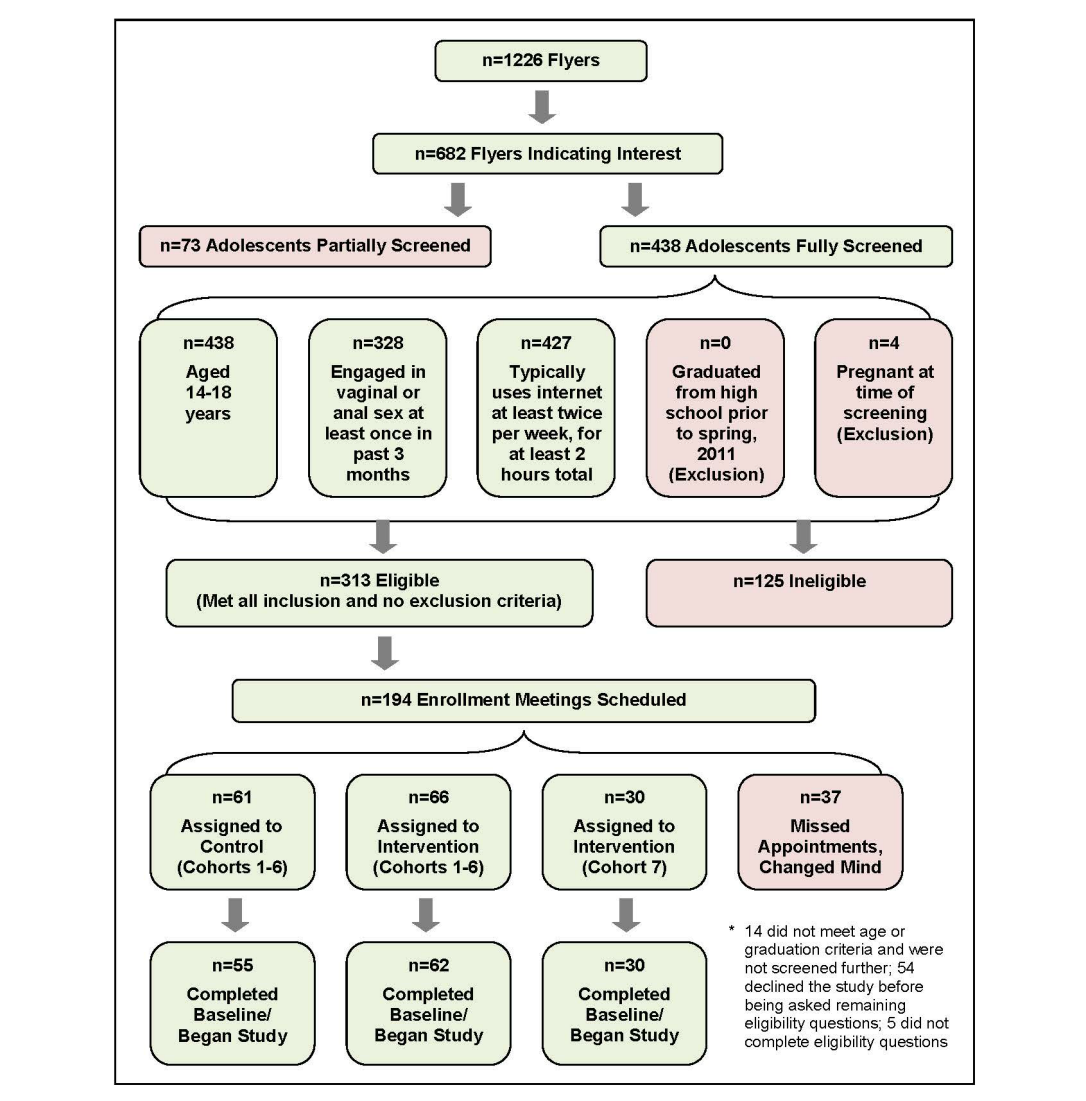
Numbers of adolescents at different stages of recruitment, screening, and enrollment.

Figure 2 depicts numbers of adolescents at different stages of recruitment, screening, and enrollment, as well as study inclusion and exclusion criteria. A total of 1226 flyers were collected across the period of recruitment. Of collected flyers, 682 indicated that an adolescent had interest in the study; 438 of the 682 adolescents were fully screened by telephone and 313 were determined to be eligible. Inclusion criteria were as follows: aged 14 to 18 years, engaged in vaginal or anal sex at least once during the past 3 months, and spent at least 2 days using the Internet independently during a typical week for a total of at least 2 hours. Adolescents who graduated from high school before spring 2011 or who were pregnant at the time of screening were ineligible. At the end of screening, adolescents aged 14 to 17 years were told that parental consent was required for participation. Study staff offered to speak directly with parents and guardians or to send a letter of introduction if the adolescent desired. Both the telephone script and letter of introduction contained an explanation that the TeensTalkHealth website was developed to “promote healthy decision making about relationships and sexual health” and that the website would feature “information and discussions about things like saying no to sex, preventing pregnancy and STIs, using condoms and other birth control methods, and signs of healthy and unhealthy dating relationships.” Enrollment meetings were scheduled with 194 adolescents who remained interested in the study and their parents if adolescents were aged 14 to 17 years; of this number, 37 adolescents were eventually not enrolled due to missed appointments, cancellations, and/or a decision not to participate.

Enrollment meetings were held in public places. Staff described the study in detail and answered questions, obtained assent and/or consent, revealed the adolescent’s study condition, provided handouts to parents and/or adolescents about Internet safety and privacy, showed sample pages from the TeensTalkHealth website (tailored to study condition), and requested privacy if a parent or legal guardian was present so that the adolescent could select a nonidentifying username, a password that met University of Minnesota Office of Information Technology requirements, and answers to password recovery questions. At the end of the meeting, staff reviewed activities that were required for reimbursement and how to contact the study team with questions. From this point forward, research staff only interacted with adolescents via the TeensTalkHealth website and private channels of communication (eg, cell phone, email, letter).

Seven successive cohorts were screened, enrolled, and introduced to the website at the beginning of a given month. Across the first 6 cohorts, 127 participants were enrolled and assigned to the intervention or control condition (Figure 2). To augment the amount of data available to evaluate intervention content, 30 additional participants were enrolled and assigned to the intervention condition as part of a seventh cohort. Ten of 157 enrolled participants failed to complete a baseline survey. Data from 92 participants assigned to the intervention condition and 55 participants assigned to the control condition are presented in this paper.

A cohort’s study involvement consisted of a preintervention period (ie, time between enrollment and the start of the next month), a 4-month intervention period, and a 2-month follow-up. All study participants were asked to complete 7 private monthly surveys online, including a baseline survey. Participants were reimbursed US $10 per survey and received a US $30 bonus if they completed all 7 surveys. As an engagement tool, control group participants who completed the first and/or second 3 surveys were additionally entered into 1 to 2 raffles for a US $20 bonus; chances of winning were 1 in 3. For the intervention group, 15 assigned intervention tasks were due on the final day of months 1 to 4. Participants who completed all tasks during a given month were reimbursed US $10; US $5 was provided for completion of 8 to 14 tasks. Thus, both intervention and control group participants could earn a maximum of US $140 across the study period.

### Website Development

Textbox 1 summarizes the website development goals. Before the pilot study, the research team consulted with youth advisors: adolescents aged 14 to 18 years and college undergraduates hired through community and university organizations focused on sexual health. Video vignettes, teen-friendly articles, and other website content were initially developed by 2 of the authors (SSB and AJK); content was iteratively refined by the research team and youth advisors during 14 group meetings held across a 5-month period. During group meetings, advisors provided suggestions on the website name, topics for website content, messages that may motivate adolescents to use condoms and engage in other health protective behaviors, moderation of website comments by health professionals, editing of video vignette screenplays, content of discussion questions, recruitment strategies, and criteria for reimbursement of participants’ engagement in study activities. Separate from group meetings, advisors were asked to complete the baseline survey (without handing back their responses to questions), provide their completion time and thoughts on the overall length and breadth of the survey, and critique the wording of survey questions and responses.

**Website development goals.**

Dose received (exposure)

1. Promote a high level of engagement on the website.
2. Encourage condom use through website content.
3. Promote a culture in which condom use is perceived as normative within different types of sexual relationships.
4. Be responsive to perceived barriers to condom use and other sexual health/relationship concerns.

Dose received (satisfaction)

1. Maximize the comfort of adolescents using the website.
2. Protect the privacy of adolescents using the website.
3. Design features that enhance the accessibility and ease of use of different sections of the website.
4. Enhance the perceived personal relevance of health communications on the website.
5. Enhance the perceived credibility of individuals delivering health communications.
6. Demonstrate respect for adolescents’ autonomy.

### TeensTalkHealth Intervention

TeensTalkHealth adopted several broad strategies to achieve website development goals. First, the website intervention featured moderated discussion between adolescent website users, whose identities were protected through nonidentifying usernames, and health educators on the research team. Website discussion on message boards evolved in response to relationship concerns and barriers to condom use identified by adolescents over time.

Second, the website featured different types of content that served as conversation catalysts between adolescents and health educators. Twenty video vignettes of young role models provided sexual health and risk-reduction information, motivation to engage in health protective behavior, and behavioral skills to negotiate condom use with partners or to address other barriers to healthy relationships. Multimedia Appendix 1 contains titles, synopses, learning objectives (not shown to participants), and discussion questions for vignettes. Ten videos addressed condom use, including planning for condom-protected sex, advocating for condom-protected sex, and handling consequences of unprotected sex. Six videos addressed setting sexual boundaries; 4 addressed coping with difficult relationship situations. Multimedia Appendices 2 and 3 contain sample video vignettes. Content was diverse to engage adolescents and address an array of factors that may affect condom use and other healthy decision making. In addition to video vignettes, teen-friendly articles and brief discussion topics also served as conversation catalysts.

A third feature of the website was easily navigable archives of video and text. The home page of the TeensTalkHealth website included Highlighted Topics, Recent Comments, and Replies to My Content boxes, and a Create New Discussion button and a featured poll. Separate pages of the website listed all titles of video topics, article topics, and other discussion topics, along with a corresponding synopsis of the topic or extract from discussion content. Adolescents could click on a specific topic to access message board discussion related to that topic. Indentation and placement of comments on message boards allowed website users to understand when a comment was made in direct response to another user, which facilitated the tracking of different conversations under a single topic. Other pages displayed all recent topics and comments, a list of resources (including emergency contact information), and a Contact Us form. Under the My Account page, website users could change their password and review currently assigned tasks, all comments and discussion topics they had submitted to that point, and replies to their content. Tabs at the top of the TeensTalkHealth website allowed users to navigate between different pages. A search bar was also present at the top of every page.

Adolescents in the intervention condition accessed website content for a 4-month period. Although users were free to access any content that was available from the time they joined, health educators assigned standard weekly content through a section of the website, My Required Tasks. Adolescents were asked to complete a total of 60 tasks across the 4-month intervention. Adolescents completed a brief private survey and public comment for each of 20 video vignettes, resulting in 40 video-related tasks. Similarly, adolescents completed a brief private survey and public comment for each of 4 teen-friendly articles, resulting in 8 article-related tasks. Finally, adolescents were asked to provide a public comment on 12 message boards with no associated video vignette or article. These “discussion-only” message boards typically began with thought-provoking information and questions posed by health educators.

To promote task and monthly survey completion, 3 to 13 reminders were sent to each participant per month through texting, voicemails, emails, and mailed letters. Targeted communications were made to those adolescents who had not yet completed tasks and/or responded to previous communications by staff. Of note, none of the adolescents complained about the frequency with which they interacted with staff.

### Moderation of Website Comments

The manner in which health educators engage adolescents is critical to the success of TeensTalkHealth and similar interventions. Key principles of TeensTalkHealth moderation included (1) demonstrating that it is possible to protect health while also establishing, maintaining, and strengthening relationships and (2) developing a climate in which adolescents feel comfortable disclosing their own experiences, sharing what they have learned, and providing guidance to others. By adding comments to video, article, and discussion topics, adolescents are able to clarify their values and beliefs. Health educators attempt to reinforce health-promoting attitudes and behaviors and respectfully challenge risk-promoting attitudes and behaviors.

During the intervention period, all submitted comments by adolescents were read at least daily and approved by health educators before they appeared publicly. When enrolled, adolescents were told that identifying information and abusive language directed toward other website users would be removed. Identifying information was rarely submitted; abusive language was never submitted. No other censorship of adolescents’ comments was made. Health educators identified and presented challenging website comments at weekly moderation meetings. Possible responses were considered by the team, which included the first author (SSB), a clinical psychologist. Moderation meetings yielded several guidelines for responding to comments:

1. Offer thought-provoking, yet specific, prompts to continue discussion.
2. Highlight adolescents’ personal strengths.
3. Praise self-awareness and, when applicable, ask for additional information about thoughts and feelings that drive decision making and behavior.
4. Provide motivation (explicit rationales for engagement in health protective behavior) and cognitive-behavioral skills (explicit strategies to engage in health protective behavior) whenever possible.
5. Reframe and challenge risk-promoting statements—try to acknowledge or validate the essence of what has been said so that adolescents will be open to “hearing” a caution against risk.
6. Empathize with stressors (acknowledge difficulty) and, when applicable, provide cognitive-behavioral skills for coping.
7. Emphasize adolescents’ autonomy and choice with respect to behavior—foster a sense of agency.
8. Challenge the idea that it is possible to completely avoid negative experiences when choosing to engage in risk.
9. Encourage adolescents to think about how past negative experiences can inform healthy decision making in the future.
10. Encourage adolescents to plan ahead—foster a sense of intentionality.

The following exchanges illustrate how health educators used moderation guidelines to address the challenge of negotiating condom use with a male partner when it is known that the female partner is using a hormonal contraceptive to prevent pregnancy. Comments have not been edited for spelling or grammar; clarifying information has been added within brackets.

I started BC [birth control] a couple of months ago. The guy I was dating at the time was super excited that he didn’t have to use a condom anymore. I mean, I wasn’t as worried about not using one [referring to a character in Video 4, see Multimedia Appendix 1], but after I always freaked out a little. As much as I know it’s bad, I didn’t make him use a condom because it made him happy. I know he would have if I had asked, but I never did. Looking back, I wish I had made him. I regret not using one, even though I didn’t get pregnant. [Multimedia Appendix 1]

Teen 1

In relationships, there is a lot of give and take and most good relationships need compromise. On the other hand, condom use and pregnancy/STI prevention is one of those places I personally think it’s okay to take a stand when your intent is to be as safe as possible. Teen 1, have you thought about how you’ll do things differently in your next relationship? What might you say to a new partner? Does anyone have suggestions about what’s worked for them?

Health Educator 1

Actually, things with that guy didn’t work out and I have a new partner. We’ve already talked about sex and condoms and all that, even though he’s abstinent. We’ve come to the conclusion that if we ever do, condoms will always be used. We both have our whole lives ahead of us, and as much as we both want kids some day, not before we can figure out our place in life. It’s nice actually, not having to stress about sex and if the condom/pill worked.

Teen 1

I completely relate to this situation [referring to Video 4, see Multimedia Appendix 1], i just wish i had enough confidence to speak to my partners like this. Its so hard to change things once theyve been happening that way for so long. Its unfortunate but i dont know how to really change it sometimes. [Multimedia Appendix 1]

Teen 2

Hey Teen 2, building your confidence can be a challenge. Some people might find it helpful to practice in front of a mirror or with a friend. Practicing saying the words out loud and many times is a really helpful way to prepare for talking about a difficult subject with a partner. Another helpful exercise to build your confidence is to make a list of reasons why what you are proposing is reasonable and desirable. If you really believe that what you want IS important for both of you, you will have an easier time staying confident in a tough conversation.

Health Educator 2

The following exchanges illustrate how health educators used moderation guidelines to address sexual behavior in the context of substance use:

i have no problem having sex with my boyfriend if hes under the influance. i think it just makes things more interesting and exciting.

Teen 3

It’s true that having sex under the influence can make the experience more unpredictable. For some people that’s exciting, but it can also be risky. Drinking or using drugs affects decision making, and can make it difficult to communicate clearly about what you want or don’t want. What are some things that people can do to make situations like this less risky? Are there other ways to make sex exciting?

Health Educator 1

i think having sex with a bofriend that has been drinking is discusting because for one their breath stinks & for two the person is not in its right state of mind, and its not ok to take advantage of them that way!

Teen 4

I agree even in a relationship where i’m comfortable having sex i have always told my boyfriend that if either one of us or both of us have been drinking sex is not an option.

Teen 5

Way to set a boundary, Teen 5! Sometimes setting up expectations and boundaries about sex before the situation happens makes it easier and less stressful in the moment.

Health Educator 2

Lastly, the following exchanges illustrate how health educators used moderation guidelines to address potentially unhealthy relationships and encourage adolescents to clarify their boundaries with respect to the acceptability of a partner’s behavior:

Can jealousy and anger be signs of love? Why or why not? If you’re not sure, what are some reasons that it is hard to decide?

Health Educator 1

I think there is a thin line, because jealousy can mean someone really cares about you, but it can also mean there over protective, it all depend on the circumstances.

Teen 6

Could you say more about what circumstances you think jealousy shows caring, and what circumstances you think jealousy crosses the line? How would a person know when to be worried about their relationship?

Health Educator 1

its sort of hard to explain, but i did read in a Cosmo magazine one time that a little bit of jealousy shown from your partner is okay. But i think it crosses the line if you’re hangingout with your friends and your partner gets jealous and angry because they see you having a good time without them. you need to keep in mind that we are all human and we all need social needs, and if we’re with the same people for ever then it gets boring, pllus you might lose a lot of friends if you’re not hangingout with them. And your friends won’t call you or invite you to things because they think you’re always with your partner. –Trust me i’ve been there and it sucks.

Teen 7

i agree with you. A little jealousy is good but it starts becomming a problem when your partner gets mad at you when your with friends and maybe not texting him/her back right away. thats too far. but at the same time, that means that the trust isnt there either. Why else would he/she be constantly asking what your doing?

Teen 8

### Categorizing the TeensTalkHealth Intervention According to Behavior Change Techniques

Michie and colleagues [54] have defined behavior change techniques—or “active ingredients”—as observable, replicable, and irreducible components of an intervention designed to alter or redirect causal processes that regulate behavior. They highlight several benefits of using a standardized taxonomy to classify the active ingredients of behavior change interventions: (1) contribution toward a comprehensive list of behavior change techniques, which can serve as a resource to others; (2) faithful implementation of interventions found to be effective; (3) accurate replication of interventions in comparative effectiveness research; (4) facilitation of systematic literature reviews and meta-analyses testing the contribution of different behavior change techniques; and (5) greater ability to link behavior change techniques to theories of behavior change and to gain insight into mechanisms of action. TeensTalkHealth uses the following behavior change techniques in Michie et al’s [54] taxonomy: considering the consequences of behavior (eg, health, social, emotional), shaping of knowledge (eg, identifying the antecedents of behavior, rehearsing how to perform a behavior), identifying goals and planning (eg, problem solving and other forms of coping, action planning, including formation of implementation intentions), providing social support (eg, emotional, informational, appraisal), comparing one’s own behavior to others through modeling (eg, video vignettes) and social comparisons (eg, teen comments on message boards), and examining one’s identity (eg, identification of the self as a role model, self-affirmation, visualization of oneself with changed behavior, reframing, addressing cognitive dissonance).

### Measurement of Process Evaluation Components

#### Overview

Sources of process evaluation data included adolescents’ responses on monthly surveys, staff experience, and automated tracking of website activity, including task and monthly survey completion.

#### Dose Received/Exposure

Different domains of intervention exposure were evaluated. First, automated tracking of website activity included (1) number of completed assigned tasks, (2) number of played and completed videos, (3) number of assigned articles and discussion topics visited, (4) number of website visits, (5) cumulative hours spent on the website, and (6) number of comments made. Completion of assigned tasks is arguably the strongest index of exposure because participants had to reflect on intervention materials to complete a brief survey or add a comment.

Second, responses on monthly surveys yielded a perceived engagement composite, the mean of 7 items: “Over the last month when you visited the website, how much interest (0=no interest, 1=a little, 2=a lot) did you have in (1) watching new videos; (2) taking private surveys about videos; (3) reading articles; (4) posting new discussion topics; (5) reading what other teens have to say about videos, articles, and discussion topics; (6) reading what health educators have to say about videos, articles, and discussion topics; and (7) responding to comments from other people to continue a discussion?”

Third, monthly surveys were used to assess participants’ perceptions of 3 website goals indicative of exposure: (1) encouragement of condom use, (2) normativeness of condom use, and (3) responsiveness to relationship concerns and barriers to condom use. Perceived encouragement of condom use was determined by calculating the mean of 5 items: “How much (1=not at all, 5=very) was the website trying to encourage consistent condom use (1) with new partners, (2) with long-term partners, (3) with casual partners, (4) with serious partners, and (5) even if someone is using hormonal contraceptives (eg, birth control pills, the patch, the shot)?” Perceived normativeness of condom use, assessed at the end of months 2 and 4 only, was determined by calculating the mean of 6 items: “How many teens on this website (1=almost nobody, 5=almost everybody) seemed to use condoms when (1) they had sex with new partners, (2) they had sex with long-term partners, (3) they had sex with someone they thought of as casual, (4) they had sex with someone they thought of as serious, (5) they were already using a hormonal contraceptive (eg, birth control pills, the patch, the shot) to keep from getting pregnant, and (6) they were using no other form of birth control?” Perceived responsiveness was determined separately for other adolescents on the website and for health educators. Perceived responsiveness was determined by calculating the mean of 3 items: “When I talked about something that keeps me from using condoms, other teens (health educators) on this website (1) said things to try to help me use condoms” and “When I talked about a problem I was having with a relationship, other teens (health educators) on this website (2) showed they cared and (3) tried to help solve the problem.” Participants rated perceived responsiveness on a 5-point Likert scale (1=not at all true, 5=very true).

#### Dose Received/Satisfaction

Monthly surveys were used to assess participants’ perceptions of 6 website goals related to satisfaction: (1) comfort, (2) privacy, (3) accessibility/ease of use, (4) personal relevance of health communications, (5) credibility of individuals delivering health communications, and (6) respect for autonomy by health educators.

Perceived comfort was determined by calculating the mean of 2 items: “When you were on the website, how comfortable (1=not at all, 5=very) have you felt (1) asking questions and (2) sharing your experiences?” Perceived privacy, assessed at the end of months 1 and 3 only, was determined through a single item (subsequently reverse-scored): “How worried (1=not at all, 5=very) are you that people not connected to this study will find out personal information you have shared on the website?” Perceived accessibility/ease of use, assessed at the end of months 1 and 3 only, was determined by calculating the mean of 6 items: “How easy (0=not at all, 1=a little, 2=very) was it to use different parts of the website: (1) watching videos, (2) taking private surveys about videos, (3) posting a new discussion topic, (4) searching the discussions for a specific topic, (5) moving from 1 part of the website to another, and (6) using the website to take monthly sexual health surveys?”

Perceived personal relevance of health communications was determined by calculating the mean of 3 items: “How much (1=not at all, 5=very) did (1) people in the videos talk about things that matter to you, (2) teens on the website talk about things that matter to you, and (3) health educators on the website talk about things that matter to you. Perceived credibility, assessed at the end of months 2 and 4 only, was determined separately for adolescents on the website, models in videos, and health educators. Perceived credibility of adolescents and models were each determined through a single item: (1) “How much (1=not at all, 5=very) did adolescents on the website (the people in videos) know what they were talking about?” This item was also assessed for health educators. In addition to this item, 3 other items [55] were assessed to calculate a 4-item mean for perceived credibility of health educators: (2) “How believable was the information from health educators?” (3) “How accurate was the information from health educators? and (4) “How trustworthy were health educators?” Perceived respect for autonomy by health educators, assessed at the end of months 2 and 4 only, was determined by separately examining 3 items: “How much (1=not at all, 5=very) were health educators (1) trying to get you to do what they want, (2) trying to help you do what you want, and (3) leaving out information to get you to do what they want?”

The internal consistency (alpha) of each multi-item measure was calculated as an index of reliability.

## Results

### Recruitment

Demographic characteristics for adolescents who returned flyers, completed the baseline survey, and completed the final (2-month follow-up) survey are shown in Table 1. In comparison to adolescents who completed recruitment flyers, the final study sample was less likely to be aged 14 to 17 years than 18 years and more likely to be female than male; ethnic diversity was similar. Nearly 42% (61/147, 41.5%) of study participants reported consistent condom use during the 3 months before screening. No differences in age, sex, ethnicity, and condom use consistency at screening were observed by study condition among the first 6 cohorts (data available on request).

**Table 1:** Demographic characteristics of adolescents who returned flyers, completed the baseline survey, and completed the 2-month follow-up survey (N=1226).^a^

Demographic characteristic		Adolescents, n (%)		
		Returned flyers (N=1226)	Completed baseline (n=147)	Completed 2-month follow-up (n=111)
**Age at baseline**				
	14-17 years	805 (72.1)	89 (60.5)	65 (58.6)
	18 years	312 (27.9)	58 (39.5)	46 (41.4)
**Sex**				
	Female	905 (80.7)	132 (89.8)	105 (94.6)
	Male	216 (19.3)	15 (10.2)	6 (5.4)
**Race/ethnicity**				
	Non-Hispanic white	729 (65.6)	92 (62.6)	70 (63.1)
	>1 race/ ethnicity	129 (11.6)	24 (16.3)	22 (19.8)
	Black/African American	133 (12.0)	16 (10.9)	10 (9.0)
	Asian or Pacific Islander	58 (5.2)	8 (5.4)	5 (4.5)
	Hispanic or Latino	50 (4.5)	5 (3.4)	3 (2.7)
	Other race/ethnicity	13 (1.2)	2 (1.4)	1 (0.9)
**Consistency of condom use in past 3 months at screening** ^b^				
	100%	n/a	61 (41.5)	49 (44.1)
	Less than 100%	n/a	86 (58.5)	62 (55.9)

^a^ Percentages are shown for those adolescents who provided data for a particular demographic characteristic on the recruitment flyer.

^b^ Consistency of condom use was not assessed until screening.

### Dose Received/Exposure

On average, intervention participants logged on 20 times, spent a cumulative 6.2 hours on the website, and submitted 24.8 comments (Table 2). The mean number of videos participants initiated and completed playing was 12.4 and 10.3, respectively. On average, intervention participants visited 2.7 assigned articles and 8.2 assigned discussion topics. Approximately one-quarter of intervention participants completed all 60 assigned tasks during the 4-month intervention period (24/92, 26%). An additional third completed 40 to 59 tasks (30/92, 33%). Less than 10% completed no tasks (8/92, 9%). Rates of task completion declined over the course of the intervention period.

**Table 2:** Distributions of website activity variables.^a^

Index of website activity			Intervention participants (n=92)	Control participants (n=55)
**Task completion, n (%)**				
	**All months**			
		0	8 (8.7)	
		1-19	15 (16.3)	
		20-39	15 (16.3)	
		40-59	30 (32.6)	
		60	24 (26.1)	
	**Month 1**			
		0	13 (14.1)	
		1-4	4 (4.3)	
		5-9	10 (10.9)	
		10-14	4 (4.3)	
		15	61 (66.3)	
	**Month 2**			
		0	16 (17.8)	
		1-4	7 (7.8)	
		5-9	10 (11.1)	
		10-14	7 (7.8)	
		15	50 (55.6)	
	**Month 3**			
		0	21 (23.6)	
		1-4	9 (10.1)	
		5-9	6 (6.7)	
		10-14	8 (9.0)	
		15	45 (50.6)	
	**Month 4**			
		0	27 (30.7)	
		1-4	8 (9.1)	
		5-9	9 (10.2)	
		10-14	5 (5.7)	
		15	39 (44.3)	
**Website interaction, mean (SD)**				
	Number of website visits^b^		20.0 (12.2)	10.1 (3.2)
	Cumulative hours spent on website^b^		6.2 (3.6)	1.7 (0.6)
	Number of comments made on website^c^		24.8 (15.3)	
	Videos with initiated play (of 20)		12.4 (7.0)	
	Videos with completed play (of 20)		10.3 (6.5)	
	Assigned articles visited (of 4)		2.7 (1.5)	
	Assigned discussion topics visited (of 12)		8.2 (4.3)	
**Monthly surveys, n (%)**				
	Month 1		75 (83.3)	53 (96.4)
	Month 2		72 (80.9)	50 (90.9)
	Month 3		66 (75.0)	53 (96.4)
	Month 4		57 (65.5)	51 (94.4)

^a^An enrolled adolescent had to complete a baseline survey to become a participant. Across the tasks shown within all months or a given month, numbers tally to the total number of participants assigned to the intervention condition (minus any withdrawn participants for months 2-4) and percentages tally to 100%.

^b^ A session “timed out” if participants did not navigate to or refresh a webpage within 15 minutes, necessitating a new visit if the participant still wanted to use the website. If participants did not log out, the timestamp for the last visited webpage was used to calculate the amount of time spent on the website during a given visit. Three outliers were not included when calculating the mean and standard deviation for cumulative hours spent on the website: 1 control group participant whose time amounted to 26.9 hours and 2 intervention group participants whose time amounted to 29.4 and 74.7 hours, respectively.

^c^ A total 36 comments were requested as part of assigned tasks.

Adolescents reported moderate levels of perceived engagement (interest) in various website activities on average (Table 3). When individual items in the perceived engagement composite were examined, 40% to 50% of participants reported high levels of interest in reading what health educators and other adolescents had to say about videos, articles, and discussion topics. On average, participants perceived that the website was strongly encouraging condom use across a variety of situations. Most participants perceived condom use to be normative among at least half of adolescents on the website. Health educators were perceived to be more responsive to adolescents’ relationship concerns than were other adolescents on the website.

**Table 3:** Participant responses to monthly survey items designed to evaluate website development goals and internal consistency (alpha) of composite measures.^a^

Construct			Month 1		Month 2		Month 3		Month 4	
			α	Mean (SD)	α	Mean (SD)	α	Mean (SD)	α	Mean (SD)
**Dose received (exposure)**										
	Perceived engagement (scale 0-2)^b^		.55	1.18 (0.30)	.67	1.17 (0.36)	.74	1.03 (0.39)	.57	1.14 (0.31)
	Encouragement of condom use		.93	4.52 (0.83)	.88	4.63 (0.56)	.85	4.59 (0.58)	.92	4.39 (0.85)
	Condom use normative^c^		—	—	.75	3.51 (0.57)	—	—	.83	3.61 (0.74)
	**Responsiveness to barriers**									
		Teens on website	.77	2.56 (1.09)	.80	2.67 (1.15)	.75	2.62 (1.09)	.79	2.79 (1.12)
		Health educators	.80	3.16 (1.20)	.82	3.35 (1.10)	.71	3.30 (1.07)	.76	3.46 (1.05)
**Dose received (satisfaction)**										
	Comfort on website^d^		.65	4.21 (0.86)	.66	4.24 (0.81)	.67	4.08 (0.89)	.61	4.01 (0.94)
	Perceived privacy		n/a	4.81 (0.51)	—	—	n/a	4.83 (0.63)	—	—
	Accessibility/ease of use (scale 0-2)^b^		.59	1.75 (0.28)	—	—	.70	1.77 (0.30)	—	—
	Personal relevance of content		.71	3.37 (0.72)	.73	3.35 (0.72)	.82	3.37 (0.89)	.77	3.42 (0.77)
	Credibility of teens on website^c^		—	—	n/a	3.50 (0.80)	—	—	n/a	3.49 (0.81)
	Credibility of people in videos^c^		—	—	n/a	4.24 (0.81)	—	—	n/a	4.15 (0.85)
	Credibility of health educators^c^		—	—	.85	4.52 (0.67)	—	—	.77	4.53 (0.64)
	Respect for autonomy by health educators^c^									
	**How much were health educators...**									
		Leaving out information to get you to do what they want?	—	—	n/a	1.64 (1.26)	—	—	n/a	1.70 (1.19)
		Trying to get you to do what they want?	—	—	n/a	2.93 (1.12)	—	—	n/a	2.85 (1.24)
		Trying to help you do what you want?	—	—	n/a	3.76 (0.94)	—	—	n/a	3.79 (0.87)

^a^ Responses are presented by end-of-month survey, collapsing across cohort. Dashes indicate that a construct was not assessed as part of a particular survey. When a single item was used to assess a construct, n/a (for not applicable) is indicated in lieu of the internal consistency (alpha).

^b^ Perceived engagement and accessibility/ease of use were assessed using a 3-point Likert scale (0-2). Other constructs were assessed using a 5-point Likert scale (1-5).

^c^ Participants were asked to think across the past 2 months.

^d^ The correlation between these 2 items is presented instead of the internal consistency (alpha).

### Dose Received/Satisfaction

Table 3 shows that participants felt a high level of comfort and perceived very high levels of privacy on the website. Mean ratings of accessibility/ease of website use approached “very easy.” Personal relevance of content and perceived credibility of other adolescents on the website were normally distributed around mean values slighter greater than these scales’ midpoints. Participants perceived high credibility of health educators and the models in video vignettes. Perceived respect for autonomy by health educators was assessed with 3 items. The perception that health educators were deliberately leaving out information was rare. Perceptions that health educators were trying to “get you to do what they want” and trying to “help you do what you want” were more common, with mean responses slightly less than and somewhat greater than the midpoints of the respective scales.

### Retention

Retention rate, assessed by monthly survey completion, varied by study condition (Table 2). More than 80% (75/90, 83%) of intervention group participants completed their month 1 survey, whereas 66% (57/87) completed their month 4 survey. Corresponding percentages among control group participants were 96% (53/55) and 94% (51/54). (Note: 5 intervention group participants and 1 control group participant had withdrawn by the time of the month 4 survey.)

## Discussion

This work demonstrates the feasibility and acceptability of TeensTalkHealth, a Web-based intervention designed to promote condom use and other healthy decision making in the context of romantic and sexual relationships. Key findings involving process evaluation components, primary challenges encountered by staff, and proposed solutions are discussed subsequently.

Adolescents’ perceptions that the website encouraged condom use across a variety of relationship situations were very high. Thus, TeensTalkHealth succeeded in its first and foremost website development goal. Most participants also perceived that condoms were used by at least half of adolescents on the website. Mean values for personal relevance of website content, including message board discussion, were greater than the scale midpoint. Adolescents’ perceptions of engagement (interest) and health educators’ responsiveness were at these scales’ midpoints, on average, whereas perceptions of peer responsiveness were less than the scale midpoint. Different strategies could be used in future applications of TeensTalkHealth to enhance engagement and perceived responsiveness. For example, tailoring may increase perceived personal relevance of intervention content, which may serve to enhance engagement, perceived responsiveness to concerns, and receptivity toward persuasion [56,57]. Assigned or recommended content could be tailored based on a small number of target areas identified through adolescents’ responses to a baseline survey and issues of concern that emerge across the intervention period. During the TeensTalkHealth intervention, health educators addressed a variety of issues that could be applied toward the tailoring of content (eg, perceived norms that stigmatize possession of condoms by girls, perceived incompatibility between condom use and trust/intimacy in relationships, greater concern for pregnancy prevention than STI prevention, lack of sexual agency, use of substances to allay sexual anxieties or to generate excitement). Allowing adolescents to schedule a one-on-one website chat with health educators, if desired, may further enhance perceived responsiveness. Similarly, holding regular live website chats between adolescents and health educators (with a slight time delay to remove any identifying information) may cultivate a greater sense of community and support among adolescents. Features of popular social networking websites could be incorporated to a greater degree within the context of TeensTalkHealth. For example, adolescents could be allowed to build their own “identity” pages, with moderation of submitted material to preserve anonymity. In addition to implementing one or more of these strategies, future applications of TeensTalkHealth should examine the extent to which responsiveness may be a function of degree of interactivity among adolescent website users and between individual adolescents and health professionals.

Almost 60% of intervention group participants completed two-thirds or more of assigned tasks across the 4-month intervention period, suggesting a reasonable level of exposure for this feasibility study. Although interest in the website and perceived responsiveness of health educators and peers may account for differences in the degree of participation, demands of the study protocol may also have been responsible. Among the intervention group, rates of task and monthly survey completion waned over time; the combination of 15 tasks and a lengthy assessment each month may have been too demanding for some adolescents. Monthly survey completion among control group participants, who had no other assigned tasks and could earn bonuses in raffles, remained greater than 90% across the intervention period. For this reason, it is recommended that assessments be conducted before and after, but not during, the intervention period. As Table 3 demonstrates, mean values for TeensTalkHealth evaluated constructs were consistent across the 4 months of intervention. Thus, assessment of constructs just after intervention completion appears reasonable and may increase participation in intervention activities. Another strategy that may enhance task completion is the incorporation of gaming features into the website [58]. For example, points could be awarded for completion of activities and messages posted in response to other adolescents; the points adolescents have accumulated could be prominently displayed on the home page with peers’ accumulated points. If desired, periodic raffles could be held; points accumulated could correspond to number of raffle entries. Raffles may be an economically feasible approach to incentivizing participation among adolescents. Most incentives in this study were for completion of lengthy monthly assessments.

The TeensTalkHealth protocol yielded high indexes of satisfaction with respect to comfort on the website, perceived privacy, website accessibility/ease of use, and perceived credibility of health educators and models in video vignettes. Mean values for perceived credibility of other adolescents were greater than the scale midpoint at both assessed time points, suggesting that adolescents could identify with and potentially learn from the life experiences of selected peers. Mean values were also greater than the scale midpoint for a key respect for autonomy item (“How much were health educators trying to help you do what you want?”). The amount of time health educators can spend developing responsive content and engaging adolescents in conversation is critical to further enhancing these indexes of satisfaction. When moderating website content, professionals could attempt to elicit healthy relationship goals from individual adolescents and provide information, motivation, and behavioral skills that will help individuals achieve articulated goals. This may further increase adolescents’ perceptions of personal relevance and respect for autonomy by health educators, which may in turn increase participation and retention.

Limitations of the present feasibility and acceptability study include underrepresentation of younger adolescents and males in the study sample. Requirement of parental consent may have been a barrier to participation for younger adolescents. If TeensTalkHealth and similar interventions are shown to reduce adolescent health risk behavior, waivers of parental consent may be considered by Institutional Review Boards of academic institutions. Similarly, health service organizations may consider website access to be an extension of services protected by privacy. Targeted recruitment (eg, through social networking sites or male-oriented organizations) may be necessary to reach higher numbers of male participants.

It is difficult to estimate the degree to which the present set of findings may be generalized to applications of TeensTalkHealth among general populations of adolescents and in contexts in which reimbursement for study activities or other incentives cannot be provided. Our convenience sample was likely comprised of adolescents who found the idea of anonymously interacting with others on a sexual health website to be particularly appealing. Additionally, adolescents may have been attracted by reimbursements for study participation. Both of these features limit generalizability of the present findings to general populations and contexts in which incentives cannot be given. It is possible that rates of participation would have been lower than that observed in the present study if incentives had not been given. The best way to determine the degree to which incentives may influence participation is to conduct future randomized controlled trials in which incentives vary across study conditions (eg, no incentives, modest raffle prizes where entries in the raffle are linked to degree of website participation, fixed reimbursement for completion of assessments and other study activities). Further, random assignment to tailored versus nontailored content across conditions of no incentives, modest incentives, and robust incentives may address the question of how to foster website engagement and participation in contexts where financial resources are limited. An additional consideration is the degree to which design-based research practices have been implemented [45]. Greater collaboration with adolescent website users and shaping of intervention content in response to adolescents’ stated needs should result in greater engagement and participation on the part of adolescents.

The TeensTalkHealth approach to health promotion is a feasible and acceptable strategy for community health practitioners and other health professionals to engage adolescents. A primary advantage of this approach is that adolescents can privately, comfortably, and candidly disclose thoughts and feelings that drive decision making. Interactive technology allows health professionals to receive immediate feedback on the helpfulness of communications, respond to potentially changing needs of adolescents over time, and continually encourage health protective behavior. As the evidence base for the effectiveness of interactive health promotion websites is being established, practitioners may use websites as a complement to existing services. TeensTalkHealth and similar interventions require an investment of time by health professionals to build relationships with individual adolescents and among adolescent website users. With the present process evaluation as an aid, practitioners in diverse settings can consider the resources needed to implement and evaluate technology-based interventions that involve moderated interaction between adolescents and health professionals.

## Appendix

Multimedia Appendix 1 Description of video vignettes designed to provide information, motivation, and behavioral skills.

Multimedia Appendix 2 Sample video vignette: No Worries.

Multimedia Appendix 3 Sample video vignette: Party Pressure.

## Abbreviations

HIV: human immunodeficiency virus
IMB: Information-Motivation-Behavioral skills
STI: sexually transmitted infections
