# Supplementary material for: An Interactive Website to Reduce Sexual Risk Behavior: Process Evaluation of TeensTalkHealth
Source: JMIR Res Protoc. 2015 Sep 2;4(3):e106. doi: 10.2196/resprot.3440 (PMC4704941; doi:10.2196/resprot.3440)
Supplement: Multimedia Appendix 1 [file resprot_v4i3e106_app1.pdf]

Video	Modeled IMB Components and Synopsis Excerpt	IMB Informed Learning Objectives	IMB Informed Sample Discussion Questions
**Month 1**			
1. Never Too Late	*Motivation and Behavioral Skills*: Marcus and Jasmine have already had sex without a condom, and now Jasmine wants to start using them. A discussion takes place about what using a condom means.	*Behavioral Skills*: • Learn ways to negotiate condom use after a couple has already had sex without condom use. • Learn ways to respond to pressure not to use condoms.	*Solicit barriers to and facilitators of health protective behaviors; Build behavioral skills*: 1. What are some reasons it's hard to bring up using condoms after two people already had sex without one? 2. Do condoms play a role in indicating the closeness of a relationship? Why or why not? 3. What are some other things Jasmine or Marcus might have said in this situation?
2. Trying Something New	*Motivation and Behavioral Skills*: Isabella and Mason are out on a date. When Mason brings up sex, Isabella suggests trying something new.	*Behavioral Skills*: • Learn ways to suggest alternatives to penetrative sex.	*Solicit barriers to and facilitators of health protective behaviors*: 1. Do you think certain kinds of sex are seen as more special or valued? Why? 2. How would you react if your partner brought a condom on a date?
3. It Didn't Seem Right	*Motivation and Behavioral Skills*: Luke had a chance to hook up with the girl he liked at a party, but he decided not to. His decision turns out even better than he could have imagined.	*Sexual Health and Risk Reduction Knowledge*: • Define healthy boundaries with respect to when to be sexual with someone.	*Build sexual health and risk reduction knowledge; Enhance motivation to engage in health protective behaviors*: 1. Do you have boundaries about the situations in which you will (or won't) have sex with someone? What boundaries do you have? 2. How can alcohol or drugs impact decisions about sex? How can they impact your ability to develop a serious relationship?
4. Keep Using Condoms	*Motivation and Behavioral Skills*: Marcus is excited to stop using condoms now that Jasmine has started taking hormonal birth control. Jasmine has a different plan.	*Sexual Health and Risk Reduction Knowledge*: • Consider the benefits of using condoms along with hormonal birth control.	*Build sexual health and risk reduction knowledge; Enhance motivation to engage in health protective behaviors; Build behavioral skills*: 1. Is it common for teens to use condoms and hormonal birth control at the same time? What are some of the benefits and challenges of using these two methods? 2. What are some things you could say to a partner to convince them to use a condom, even if hormonal birth control is already being used?

5. Not Tonight	*Motivation and Behavioral Skills*:<br>Valerie brings up having sex with the guy she's seeing and is surprised by his reaction.	*Behavioral Skills*:<br>• Learn how to decline sexual activity while maintaining a new relationship.	*Build sexual health and risk reduction knowledge*; *Build behavioral skills*:<br>1. How do you know when you are ready to have sex with a partner? How do you know that you want to wait to have sex?<br>2. When do you start a conversation about sex in a new relationship? How do you start the conversation?
**Month 2**			
6. The Morning After	*Information, Motivation, and Behavioral Skills*:<br>Jasmine and Logan have a condom accident and are worried about pregnancy. They learn about emergency contraception (EC) and decide to use it before it's too late.	*Behavioral Skills*:<br>• Learn how to address barriers to obtaining EC.<br>• Learn ways for males to support female partners and share responsibility for pregnancy.	*Solicit barriers to and facilitators of health protective behaviors*:<br>1. What would make a teen less likely or more likely to want EC if they had unprotected sex or experienced an accident, like a condom breaking?<br>2. What would make a teen less likely or more likely to tell a parent that they would like EC?<br>3. Would most guys be as supportive as Logan? Why or why not?
7. Clinic Visit, Part I	*Information and Motivation*:<br>Logan is nervous to go to the clinic for a health problem. He meets with Dr. Mills, who helps him get the services he needs.	*Sexual Health and Risk Reduction Knowledge*:<br>• Learn the typical process of a clinic visit.<br>• Learn the common procedures for STI testing.	*Solicit barriers to and facilitators of health protective behaviors; Build sexual health and risk reduction knowledge*:<br>1. What qualities would you look for in a doctor to make you feel comfortable talking about sensitive health issues?<br>2. Who should get tested for STIs? Does it depend on who a person has sex with and what they do? Does it depend on other things?
8. Clinic Visit, Part II	*Information, Motivation, and Behavioral Skills*:<br>Logan goes back to the clinic to get his results after being tested for STIs. Dr. Mills gives Logan a demonstration on using condoms correctly.	*Behavioral Skills*; *Sexual Health and Risk Reduction Knowledge*:<br>• Learn steps to use a condom correctly.<br>• Learn the importance of notifying partners when there is a positive test.	*Solicit barriers to health protective behaviors, Build behavioral skills*:<br>1. In the video, Logan is honest with Dr. Mills that he uses condoms "some of the time." What are some things you can do to make sure condoms are used all the time?<br>2. In what ways would it be difficult to notify a partner about being diagnosed with an STI? What could you do to make it easier?
9. Before It Heats Up	*Information, Motivation, and Behavioral Skills*:<br>Luke and Valerie are planning to have	*Behavioral Skills*:<br>• Learn how to communicate about condom use before sex	*Build behavioral skills*; *Solicit barriers to and facilitators of health protective behaviors*; *Enhance motivation to engage in health protective behaviors*:<br>1. What advice would you give to someone

	sex, and realize they have some things to learn in order to be safe.	happens.	who wanted to talk about using condoms with a partner? What are some fun ways to bring up this discussion?<br>2. What are some benefits to talking about condoms before you have sex? Are there any drawbacks? How often do you think people your age talk about pregnancy and STI prevention before sex?
10. I Have to Tell You Something	*Information, Motivation, and Behavioral Skills*:<br>Mason and Chloe are going to a movie, but first Chloe has something important to talk about.	*Behavioral Skills*:<br>• Learn how to disclose having a STI to a new partner, including staying calm and providing facts.	*Build sexual health and risk reduction knowledge; Build behavioral skills*; *Solicit barriers to and facilitators of health protective behaviors*; *Enhance motivation to engage in health protective behaviors*:<br>1. When should somebody first bring up an STI in a new relationship? How should they bring it up?<br>2. If you had an STI, would you tell your partner? Does it depend on the STI? Does it depend on the relationship? Why would you tell or not tell?
**Month 3**			
11. Party Pressure	*Motivation and Behavioral Skills*:<br>Marcus and Isabella are at a party and have an opportunity to be alone in an upstairs bedroom. Isabella challenges the concept of "alone" and shares her thoughts for what makes sex good.	*Behavioral Skills*:<br>• Learn how to decline sexual activity while still maintaining a long-term/established relationship.	*Build sexual health and risk reduction knowledge*; *Solicit barriers to health protective behaviors*; *Enhance motivation to engage in health protective behaviors*; *Build behavioral skills*:<br>1. Imagine that a guy or girl decides they'll only have sex if things feel right. What makes things "right"?<br>2. Do you think people sometimes have sex when they don't want to, so that they don't cause trouble in a relationship? Is this okay? Why or why not?<br>3. How can someone say "no" to sex in the moment, while still making it clear that they're interested in the other person sexually?
12. Coming Out	*Information, Motivation, and Behavioral Skills*:<br>Logan is in a relationship with Justin, and he is keeping this a secret because he's afraid to "come out." He finally decides to confide in a trusted friend.	*Behavioral Skills*; *Sexual Health and Risk Reduction Knowledge*:<br>• Learn ways in which one could respond in a supportive manner to a friend coming out.<br>• Recognize and	*Build behavioral skills*; *Build sexual health and risk reduction knowledge*:<br>1. Not all friends might be as supportive of being gay as Taylor was of Logan. What are different ways that someone could cope with negative reactions from others, including friends and family?<br>2. If a friend came out to you, what are some ways you could be supportive?<br>3. What do you think of labels such as gay, straight, and bisexual? Why might these

		describe the negative impact of discrimination based on sexual orientation.	labels be helpful, harmful, or both of these things?
13. Buying Condoms	*Information, Motivation, and Behavioral Skills*:<br>Valerie and Olivia team up to buy condoms for the first time.	*Behavioral Skills*; *Sexual Health and Risk Reduction Knowledge*:<br>• Learn how to cope with potential embarrassment when buying condoms.<br>• Learn information about how to choose condoms.	*Build behavioral skills*; *Solicit barriers to and facilitators of health protective behaviors*; *Enhance motivation to engage in health protective behaviors*:<br>1. Have you ever tried to buy condoms? How did it go?<br>2. Would you feel embarrassed to buy condoms like Olivia did? Why or why not? What are some ways people deal with the embarrassment of buying condoms?
14. But He Loves Me	*Information and Motivation*:<br>Isabella and Kayla are close friends. Isabella has some concerns about Kayla's relationship with Devan. Is she right to be concerned?	*Sexual Health and Risk Reduction Knowledge*; *Behavioral Skill*:<br>• Learn how to recognize early warning signs of dating violence.<br>• Learn how to set boundaries for a romantic and/or sexual partner's behavior.	*Build sexual health and risk reduction knowledge*; *Build behavioral skills*:<br>1. Can jealousy and anger be signs of love? Why or why not? If you're not sure, what are some reasons that it is hard to decide?<br>2. Would you feel angry if a boyfriend/girlfriend was spending time with someone you didn't like or trust? Would it be okay for a partner to be angry with you if these roles were reversed? How should anger be handled in this situation?<br>3. When does "checking in" get excessive? How would you set boundaries with a partner if you needed to?
15. Ask Me Anything	*Information*:<br>Taylor has some questions about virginity and sex. She talks it over with her friend Justin.	*Sexual Health and Risk Reduction Knowledge*:<br>• Examine values and beliefs with respect to "virginity," abstinence, and definitions of sex.<br>• Understand the importance of condoms and lubrication if one chooses to have	*Build sexual health and risk reduction knowledge; Enhance motivation to engage in health protective behaviors*:<br>1. What counts as sex and what doesn't? Would someone "lose their virginity" if they had anal sex? If they had oral sex?<br>2. How is virginity different from abstinence?<br>3. Do you think that abstinence is only about the time until someone "loses their virginity"? Can someone who has already had sex decide to be abstinent? Why might a person decide not to have sex for awhile?

		anal sex.	

Month 4

16. No Worries	*Information, Motivation, and Behavioral Skills*:<br>Marcus and Isabella have some troubles when trying to use a condom.	*Motivation to Engage in Health Protective Behaviors*; *Behavioral Skills*:<br>• Remain committed to condom use even if a partner is having difficulty using one.<br>• Show that acceptance of a partner is not conditioned on his/her sexual performance in the moment.	*Solicit barriers to health protective behaviors*, *Enhance motivation to engage in health protective behaviors*:<br>1. Why do you think a guy might be embarrassed to talk about not staying hard when he tried to put a condom on? Are there other things that can happen to a guy during sex that might make him embarrassed or frustrated? What are some benefits of talking about this with a partner? Are there drawbacks?<br>2. Girls' bodies don't always work the way they want. What are some things that can happen to a girl during sex that might make her embarrassed or frustrated? What are some benefits of talking about this with a partner? Are there drawbacks?
17. Heartbreak	*Motivation and Behavioral Skills*:<br>Sophia is talking with her friend Olivia about rumors that her boyfriend Aaron has been cheating on her. The two friends discuss different possibilities for how Sophia can confront Aaron and say what she is feeling.	*Behavioral Skills*:<br>• Consider different options and possible consequences when making difficult decisions.<br>• Learn how to role play to practice assertive communication.	*Solicit barriers to and facilitators of health protective behaviors*; *Enhance motivation to engage in health protective behaviors*; *Build sexual health and risk reduction knowledge*:<br>1. What are the risks of continuing a relationship with someone who has cheated before? Are there benefits?<br>2. With whom would you feel most comfortable role playing a conversation about cheating or a potential break up, like Sophia and Olivia did in the video? What qualities of this person would make you feel comfortable practicing a tough conversation with them?<br>3. What is cheating to you? Do you think that the type of cheating matters? For example, is there a difference between kissing and having sex with someone else? Is there such a thing as cheating "emotionally"? If so, how does this compare to cheating physically?
18. Caught Up	*Motivation*:<br>Kayla tells her older sister Faith about having sex with Jarrod for the first time. Faith asks some questions that make Kayla think about doing it	*Motivation to Engage in Health Protective Behaviors*:<br>• Consider reasons and strategies to be an active, rather than a passive	*Solicit barriers to health protective behaviors*; *Enhance motivation to engage in health protective behaviors*; *Build behavioral skills*:<br>1. What are some reasons why sex "just happens"? What are some advantages of planning ahead for sex? Are there any drawbacks?<br>2. What are some ways to communicate

	differently next time.	partner in the context of a sexual experience.	about what you want to do sexually with your partner before, during, and after sex?
19. When It's Over	*Motivation and Behavioral Skills*:<br>Chloe and Isabella discuss whether it's time for Chloe to end her relationship with Whitney.	*Behavioral Skills*:<br>• Consider how a person could end a relationship in an honest and direct way.	*Build sexual health and risk reduction knowledge; Solicit barriers to and facilitators of health protective behaviors*; *Enhance motivation to engage in health protective behaviors*; *Build behavioral skills*:<br>1. How do you know when a relationship is no longer working? How do you know when it's time to break up with someone?<br>2. Is there value to being single between relationships? Why or why not?<br>3. What are some reasons it can be hard to break up? What things can make it easier? What is the best way to end a relationship?
20. Ready or Not	*Information and Motivation*:<br>Several people share what it means to them to be ready to have sex.	*Sexual Health and Risk Reduction Knowledge*; *Motivation to Engage in Health Protective Behaviors*:<br>• Consider different aspects of being ready for sex.<br>• Determine for oneself what it means to be ready for sex.	*Build sexual health and risk reduction knowledge*; *Solicit barriers to health protective behaviors*; *Enhance motivation to engage in health protective behaviors*:<br>1. How do people know when they are ready to have sex for the first time? Does it matter if they are deciding about having sex for the very first time ever, or if it's the first time with a new partner?<br>2. Are there wrong reasons for having sex? What are they? Who decides what are the right reasons or wrong ones?
